# A continuous metal-insulator transition driven by spin correlations

**DOI:** 10.1038/s41467-021-23039-6

**Published:** 2021-05-13

**Authors:** Yejun Feng, Yishu Wang, D. M. Silevitch, S. E. Cooper, D. Mandrus, Patrick A. Lee, T. F. Rosenbaum

**Affiliations:** 1 Okinawa Institute of Science and Technology Graduate University, Onna Okinawa, 904-0495 Japan; 2 Division of Physics, Mathematics, and Astronomy, California Institute of Technology, Pasadena, CA 91125 USA; 3 The Institute for Quantum Matter and Department of Physics and Astronomy, The Johns Hopkins University, Baltimore, MD 21218 USA; 4 Department of Materials Science and Engineering, University of Tennessee, Knoxville, TN 37996 USA; 5 Materials Science and Technology Division, Oak Ridge National Laboratory, Oak Ridge, TN 37831 USA; 6 Department of Physics, Massachusetts Institute of Technology, Cambridge, MA 02138 USA

**Keywords:** Phase transitions and critical phenomena, Electronic properties and materials, Magnetic properties and materials

## Abstract

While Mott insulators induced by Coulomb interactions are a well-recognized class of metal-insulator transitions, insulators purely driven by spin correlations are much less common, as the reduced energy scale often invites competition from other degrees of freedom. Here, we demonstrate a clean example of a spin-correlation-driven metal-insulator transition in the all-in-all-out pyrochlore antiferromagnet Cd_2_Os_2_O_7_, where the lattice symmetry is preserved by the antiferromagnetism. After the antisymmetric linear magnetoresistance from conductive, ferromagnetic domain walls is removed experimentally, the bulk Hall coefficient reveals four Fermi surfaces of both electron and hole types, sequentially departing the Fermi level with decreasing temperature below the Néel temperature, *T*_N_ = 227 K. In Cd_2_Os_2_O_7_, the charge gap of a continuous metal-insulator transition opens only at *T* ~ 10 K << *T*_N_. The insulating mechanism parallels the Slater picture, but without a folded Brillouin zone, and contrasts sharply with Mott insulators and spin density waves, where the electronic gap opens above and at *T*_N_, respectively.

## Abstract (web summary)

The Mott transition is a celebrated example of a metal-insulator transition driven by electron correlations. Here, using magnetoresistance measurements, the authors demonstrate the presence of a spin-driven metal-insulator transition, where the insulating gap only opens below the Neel temperature.

## Introduction

Unlike insulators or semiconductors derived from simple metals such as sodium and lithium^1,2^, metal-insulator transitions in correlated-electron systems reside outside the paradigm of the single-electron band structure. Factors such as reduced dimensionality and randomness^3,4^ enrich the description of the critical behavior, with possible separation of spin and charge, and deep connections to exotic states such as high *T*_c_ superconductivity and quantum spin liquids^5^. In the limit of large electronic correlations, the starting point for discussion is usually the opening of the Hubbard gap^6^, with antiferromagnetic order being a subsidiary effect.

**Fig. 1:**
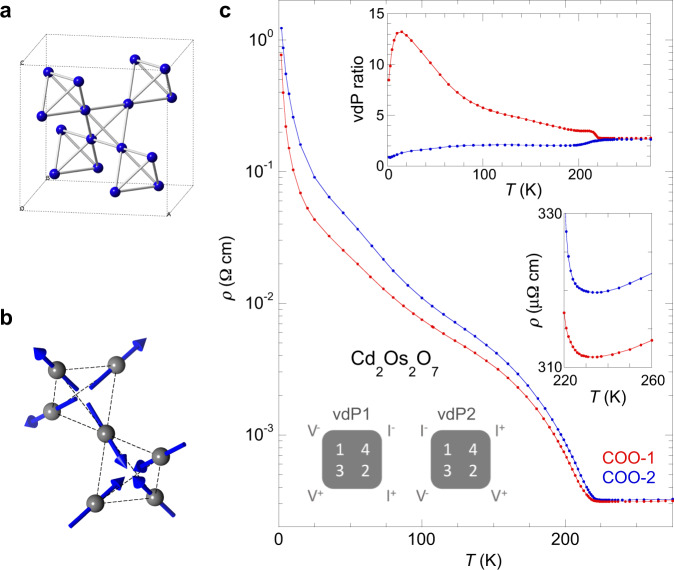
Transport signatures of Cd_2_Os_2_O_7_ at zero field. **a** Schematic of the Os sublattice in the cubic pyrochlore oxide Cd_2_Os_2_O_7_, with all Os atoms forming a three-dimensional corner-sharing octahedra network. **b** Schematic of AIAO spin arrangement on two adjacent octahedral. Note that this spin arrangement does not break the underlying lattice symmetry. **c** Resistivity *ρ*(*T*) of Cd_2_Os_2_O_7_ measured over two single crystals (COO-1 in red and COO-2 in blue) using the vdP configuration (Schematics). At each temperature, both vdP channels were measured in order to account for the changing current path between the insulating bulk and conductive domain walls. (Top inset) The resistance ratio *R*_vdP2_/*R*_vdP1_ between two vdP channels manifests the changing current path below *T*_N_ = 227K. (Bottom inset) Details of *ρ*(*T*) near *T*_N_, showing both metallic behavior above *T*_N_, and also an upturn of *ρ* near but still above *T*_N_, which is often attributed to dynamical spin fluctuation effects without long-range order. *T*_N_ is determined precisely from the magnetic susceptibility *χ*(*T*), individual *R*_vdP2_(*T*) and *R*_vdP1_(*T*) (not shown), and *R*_H_(*T*) in Fig. 3a.

There exist several major experimental challenges in establishing a convincing example of a metal-insulator transition driven by spin correlations^7^. First, the spin-correlation energy is typically much smaller than the direct Coulomb interaction, often at a scale comparable to that of the structural modifications induced by the antiferromagnetic order through magnetostrictive effects and symmetry changes, leading to a chicken-and-egg conundrum between ascribing the insulating transition to the lattice and the magnetism^8,9^. It is thus preferable to search for candidate systems in which the magnetism would preserve the crystalline symmetry. Materials that demonstrate an all-in−all-out (AIAO) antiferromagnetic order on a pyrochlore lattice, with all four spins on the corner of a tetrahedron pointing either towards or away from the center (Fig. 1a, b), meet this criterion. The AIAO order induces no symmetry-breaking magnetostriction and causes an isotropic expansion of the cubic unit cell by a minimal Δ*a*/*a* ~ 10^−4^, only becoming experimentally resolvable when *T*_N_ drops below 40 K^10,11^. For AIAO order with *T*_N_ > 100 K, this overall magnetostriction is fully camouflaged by the thermal lattice contraction^10^, which broadens the bandwidths but introduces no band splitting. The family of AIAO antiferromagnets thus provides a highly desirable model system to investigate spin correlations with little lattice interference.

Identifying an AIAO system with a metal-insulator transition represents the next experimental challenge. Accompanying the AIAO order, many 5*d* oxides, such as *R*_2_Ir_2_O_7_ (*R* = Eu, Sm, and Nd) and Cd_2_Os_2_O_7_ (ref. ^12^ and references in Refs. ^11,13,14^), also demonstrate a change of temperature dependence in the resistivity at *T* ~ *T*_N_^9,13,15,16^. However, the resistive behavior of *R*_2_Ir_2_O_7_ (*R* = Nd, Sm, Eu), especially in the paramagnetic phase, is often inconsistent and raises concerns about their intrinsic, disorder-free behavior^13,17,18^. Cd_2_Os_2_O_7_ has presented consistent behavior in both the electron correlation and metal-insulator transition. Samples from several groups^9,15,16^ always demonstrate magnetic ordering at *T*_N_ = 225–227 K, metallic behavior above *T*_N_, and a three-to-four-decade rise of the resistivity for *T* < *T*_N_ in the best samples. This repeatability from crystal to crystal likely indicates a low level of disorder because of the 2+/5+ valence condition of Cd and Os ions as well as the chemical transport growth procedure at low temperature^9,15,16^.

There exists an additional challenge arising from complications in modeling and understanding the transport data in many 5*d* AIAO antiferromagnets due to the intrinsically conductive and highly coercive ferromagnetic domain walls^9,19–21^. Ferromagnetic domain walls introduce a Zeeman shift in the metallic paramagnetic band structure, but they are not expected to gap the Fermi surface like the antiferromagnetic bulk, and they are expected to remain metallic down to *T* = 0. As was pointed out recently^21^, the highly coercive metallic ferromagnetic domain walls generate antisymmetric linear magnetoresistance (MR) of the same functional form as the Hall resistance. Moreover, the antisymmetric linear MR is detectable in Hall channels due to the distorted current paths through the domain walls^21^. This effect is likely the root cause of the widely varying Hall coefficient reported in the literature for Cd_2_Os_2_O_7_^9,16^, as the standard procedure for extracting Hall resistance through antisymmetrization with respect to magnetic field direction leads to erroneous results.

Here we present high-fidelity resistivity and Hall coefficient measurements on single-crystal Cd_2_Os_2_O_7_ after employing an intricate procedure to eliminate the influence of conductive ferromagnetic domain walls. Unlike the common understanding of a concurrent metal-insulator transition with the AIAO magnetic transition at *T*_N_, our results reveal that Cd_2_Os_2_O_7_ is metallic for a broad temperature range below *T*_N_, despite an increasing resistivity with decreasing *T*. It only becomes an insulator at *T*_MIT_ ~ 10 K, when four sets of Fermi surfaces have sequentially left the Fermi level to open a true electronic gap. This large separation in temperature for spin order (*T*_N_) and the charge gap (*T*_MIT_), with *T*_N_ >> *T*_MIT_, unambiguously establishes spin ordering as the driving force in Cd_2_Os_2_O_7_’s metal-insulator transition. Our methodology in separating the Hall behavior of the bulk from the influences of the domain walls should provide a generic approach to parsing spin and charge effects in correlated antiferromagnetic insulators with metallic domain walls.

## Results

### Insulating behavior at zero field

**Fig. 2:**
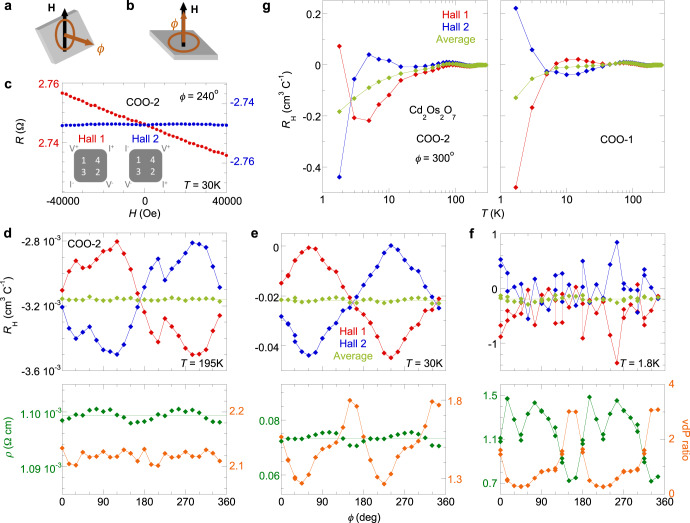
Separating the influence of metallic ferromagnetic domain walls. **a**, **b** Schematics of the measurement procedure using the two-rotator setup. Every galvanomagnetic Cd_2_Os_2_O_7_ sample (gray plate) is field-cooled in two stages: **a** field **H** is aligned parallel to the sample surface at room temperature before cooling down through *T*_N_, and **b** the sample is rotated below *T*_N_ to have a field perpendicular to its surface for galvanomagnetic measurements. The in-plane magnetizing direction is defined by angle *ϕ* at 24 discrete positions, set outside the cryostat at room temperature. **c** Typical raw data of Hall resistances *R*(*H*) between two reciprocal channels, measured for sample COO-2 at a specified temperature and magnetization angle *ϕ*. The difference in slopes indicates the influence of asymmetric linear magnetoresistance from ferromagnetic domain walls^21^. (inset) vdP configuration for the two reciprocal Hall channels. **d–f** *ϕ*-dependence of Hall resistivity slopes *R*_H_ in two reciprocal channels (red and blue) and their average (fresh green), in addition to the resistivity *ρ* (green) and vdP ratio (orange), measured in sample COO-2 at three temperatures 195, 30, and 1.8 K. (Methods) (**g**) Hall resistivity slopes *R*_H_ from two reciprocal channels (red and blue) are plotted alongside their average (fresh green) for two different single-crystal Cd_2_Os_2_O_7_ samples COO-1 and COO-2. Although the resistance slopes of an individual Hall channel are very different for each sample, with occasional crossings at various temperatures, the averages are similar in shape between the two samples and determine the bulk Hall coefficient *R*_H_(*T*). This irregular behavior of resistance slopes of individual Hall channels highlights potential experimental deficiencies in previous Hall measurements. All statistical uncertainties are smaller than the symbols.

Our single-crystal Cd_2_Os_2_O_7_ samples demonstrate a monotonic rise of resistivity over three decades (~3000×) from *T*_N_ to 1.8 K at zero field (Fig. 1c), consistent with the best samples reported in the literature^9,15,16^. Instead of using bar-shaped samples for both resistivity and Hall measurements, our key approach is to utilize the van der Pauw (vdP) configuration of electrical lead placement on plate-shaped samples (ref. ^22^, schematics in Figs. 1c, 2c). This choice of putting four leads on an equal footing aims to comprehensively evaluate the effects of the electrical current paths, which constantly redistribute between the conductive domain walls and an increasingly insulating bulk as the temperature decreases. As demonstrated by our samples, the vdP ratio, defined as *R*_vdP2_/*R*_vdP1_ at zero field, stays constant only in the paramagnetic phase above *T*_N_. Below *T*_N_, the vdP ratios of two samples have a strong temperature dependence (Fig. 1c, inset) but still evolve continuously as expected from the lack of lattice symmetry breaking in Cd_2_Os_2_O_7_. The resistivity in Fig. 1c is calculated from *R*_vdP2_ and *R*_vdP1_ at each *T* according to the standard vdP procedure^22^, an issue we will revisit below.

### Extracting the bulk Hall coefficient

The highly coercive, field-independent magnetization **M** of the conductive ferromagnetic domain walls necessarily introduces antisymmetric linear MR^21^ that makes the Hall resistances of the two reciprocal channels, *R*_12,34_(**H**, **M**) and *R*_43,12_(**H**, **M**), have different linear slopes with *H* (Fig. 2c). Due to the cubic symmetry of AIAO order, it is not possible to create a single antiferromagnetic domain and remove the domain walls altogether by field cooling. The separation of galvanomagnetic responses of the bulk domains and the domain walls is instead carried out by introducing a variable **M**(*ϕ*) through field-cooling along 24 angular directions *ϕ* within the sample surface plane (Fig. 2a, b, Methods, and ref. ^21^). Here we first examine the *ϕ*-dependences of the Hall resistivity slopes from two reciprocal channels ${R}_{{\mathrm{H}}}^{{\mathrm{Hall}}1}\left(\phi \right)$ and ${R}_{{\mathrm{H}}}^{{{\mathrm{Hall}}}2}\left(\phi \right)$, the vdP ratio, and resistivity *ρ*(*H* = 0), at temperatures 195, 30, and 1.8 K (Fig. 2d–f), where the ferromagnetic domain walls have different levels of influence as gauged by the conductance and the bulk Hall coefficient.

At all three temperatures (Fig. 2d−f), the average slopes of the two Hall resistivities, ${R}_{{\mathrm{H}}}(\phi )=[{R}_{{\mathrm{H}}}^{{{\mathrm{Hall}}}1}\left(\phi \right)+{R}_{{\mathrm{H}}}^{{{\mathrm{Hall}}}2}\left(\phi \right)]/2$, follow a constraint that (1) ${R}_{{\mathrm{H}}}(\phi )$ are always *ϕ*-independent. On the other hand, the resultant *ϕ*-dependent Hall resistivity slopes of the reciprocal channels, ${\Delta R}_{{\mathrm{H}}}^{{{\mathrm{Hall}}}{\mathrm{1,2}}}\left(\phi \right)={R}_{{\mathrm{H}}}^{{{\mathrm{Hall}}}{\mathrm{1,2}}}\left(\phi \right)-{R}_{{\mathrm{H}}}(\phi )$, behave differently. At 195 and 30 K, ${\Delta R}_{{\mathrm{H}}}^{{{\mathrm{Hall}}}1}\left(\phi \right)$ and ${\Delta R}_{{\mathrm{H}}}^{{{\mathrm{Hall}}}2}\left(\phi \right)$ follow a second constraint that (2) they are identical at *ϕ* and *ϕ* + π respectively ${\Delta R}_{{\mathrm{H}}}^{{{\mathrm{Hall}}}1}\left(\phi \right)={\Delta R}_{{\mathrm{H}}}^{{{\mathrm{Hall}}}2}\left(\phi +\pi \right)$, leading to identical *ϕ*-averaged resistivity slopes of both Hall channels. From the previous analysis^21^, constraint (1) reflects the voltage−current reciprocity, while constraint (2) manifests Onsager’s reciprocity relation with regard to the ferromagnetic domain wall **M**. At 1.8 K, the *ϕ*-dependent Hall resistivity slopes ${\Delta R}_{{\mathrm{H}}}^{{{\mathrm{Hall}}}1}\left(\phi \right)$ and ${\Delta R}_{{\mathrm{H}}}^{{{\mathrm{Hall}}}2}\left(\phi \right)$ (Fig. 2f) behave differently. Only constraint (1) is sustained, while constraint (2) cannot be satisfied by any choice of the *ϕ*-independent components. The result is that the *ϕ*-averaged resistivity slopes of individual Hall channels are no longer necessarily equal to each other and to the *ϕ*-independent component ${R}_{{\mathrm{H}}}(\phi )$.

The breakdown of constraint (2) seemingly implies a violation of Onsager’s reciprocity relation between *ϕ* and *ϕ* + π states. However, the established Onsager’s reciprocity in our system hinges on the inverse relationship between domain wall magnetizations **M**(*ϕ*) and **M**(*ϕ* + π) through separate field-cooling processes at *ϕ* and *ϕ* + π. Their inverse relationship is generally robust in that both *ρ* and the vdP ratio at zero field demonstrate a π-periodicity at all temperatures (Fig. 2d–f). At 1.8 K, the conduction patterns at *ϕ* and *ϕ* + π are similar enough to be reproducibly differentiated from those at neighboring *ϕ* positions, judging by *ρ* and the vdP ratio (Fig. 2f). Nevertheless, because of the separate cooldowns, there exist differences beyond an inversion between **M**(*ϕ*) and **M**(*ϕ* + π). With the increasingly insulating bulk, the electrical current is more concentrated along a fraction of the domain walls, as reflected by the dramatically oscillating vdP ratio from 3:1 to 1:4 (Fig. 2f). While the entire sample volume brings better averaged galvanomagnetic behavior and demonstrates Onsager reciprocity (Fig. 2d, e), probing only a small number of domain walls enhances the relative difference between **M**(*ϕ*) and **M**(*ϕ* + π). Even at a fixed *ϕ*, multiple field-coolings can lead to a significant difference in individual Hall channels at 1.8 K (Fig. 2f), despite the consistency at 30 K (Fig. 2e). We note that the assumed uniform medium for the vdP technique^22^ is justified *a posteriori* at 195 K and 30 K, as the calculated *ρ*(*ϕ*) varies within ±0.1% and ±3%, respectively. Constraint (2) remains satisfied (Fig. 2d, e). At 1.8 K, *ρ*(*ϕ*) calculated from the vdP formula^22^ varies by ±50% (Fig. 2f). Although the uniformity assumption no longer holds at 1.8 K to legitimize both the vdP-derived resistivity and constraint (2), our procedure to extract the Hall coefficient *R*_H_ through the average slope of two Hall resistivity channels is protected by the fundamental principle of voltage−current reciprocity.

**Fig. 3:**
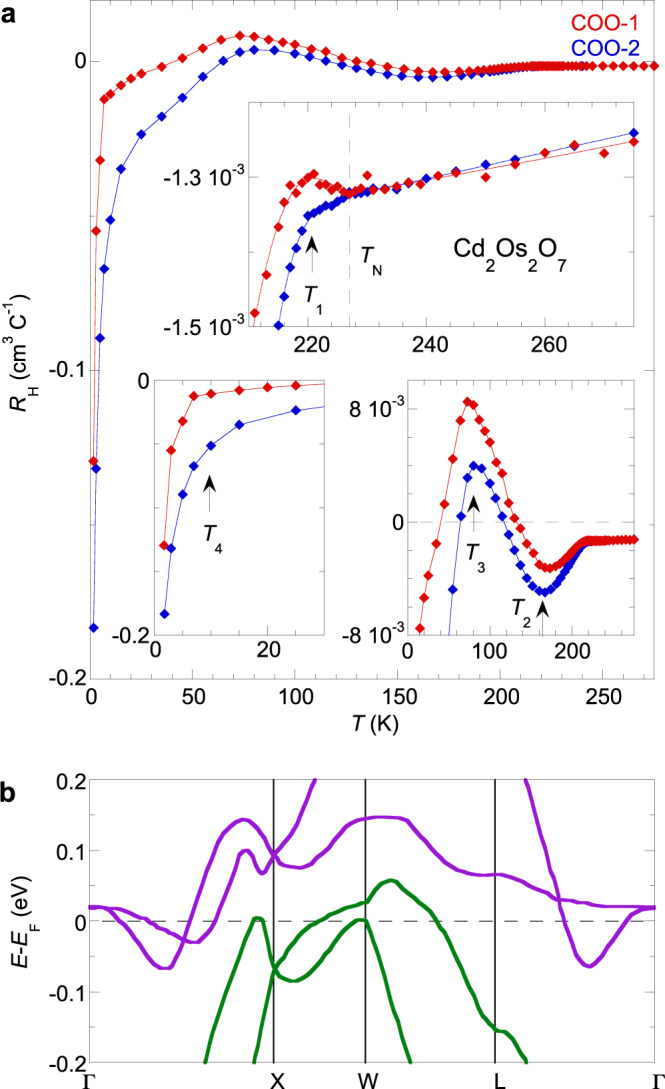
Metal−insulator transition in Cd_2_Os_2_O_7_ revealed through bulk Hall coefficient. **a** Temperature evolution of the bulk Hall coefficient *R*_H_(*T*) from two Cd_2_Os_2_O_7_ samples COO-1 and COO-2 in Fig. 2g are compared in detail. While *R*_H_(*T*) evolves slowly above *T*_N_, it starts to deviate from the high-temperature behavior right at *T*_N_, indicating that the onset of AIAO antiferromagnetic order influences the band structure around the Fermi level. Nevertheless, *R*_H_(*T*) does not diverge until it reaches a temperature below 10 K ~ 0.044 *T*_N_ when the true charge gap opens. In between, each sharp change in *R*_H_(*T*) (close-up view of the main figure in individual insets) indicates a band leaving the Fermi level, marked by arrows at four different temperatures, *T*_1_ = 220 K, *T*_2_ = 165 K, *T*_3_ = 80 K, and *T*_4_ = 10 K. Two bands are of hole type and the other two of electron type. All statistical uncertainties are smaller than the symbols. **b** Band structure of Cd_2_Os_2_O_7_ near the Fermi level. $(E-{E}_{{\mathrm{F}}})$ along trajectories between reciprocal space points $\Gamma$, X, W, and L are adapted from calculations in ref. ^26^. The four bands, two of hole (green) and two of electron (purple) types, are sequentially driven away from the Fermi level by the increasing strength of antiferromagnetic order to form a band insulator.

With the understanding of how to extract *R*_H_ through a *ϕ*-dependence study at three fixed temperatures, we now explore the temperature evolution of *R*_H_ by taking the average Hall resistivity slope of two reciprocal channels at a fixed *ϕ* (Fig. 2g). The *R*_H_(*T*) measured on two different Cd_2_Os_2_O_7_ single crystals (Fig. 3a) demonstrates a consistent picture that represents the major finding of this work. *R*_H_(*T*) remains stable above *T*_N_ = 227 K, and all bands only start to evolve at *T*_N_, maintaining a delicate balance between them until the first sharp change at 220 K. Despite a rise of nearly three orders of magnitude in *ρ*(*T*) from *T*_N_ down to 10 K, *R*_H_(*T*) remains finite and oscillates between positive and negative values. As *ρ*(*T*) reflects a fast drop in total carrier density with decreasing temperature (Fig. 1c), the oscillating *R*_H_(*T*) reflects the changes in the bands, with an alternating dominance by either electrons or holes as the major composition of itinerant carriers. In a multiband model with both electron and hole types of carriers, ${R}_{{\mathrm{H}}}\,=\,\tfrac{1}{{ec}}({\sum }_{i\in {\{h\}}}{N}_{i}{\mu }_{i}^{2}\,-\,{\sum }_{j\in {\{e\}}}{N}_{j}{\mu }_{j}^{2})/{({\sum }_{i\in {\{h\}}}{N}_{i}{\mu }_{i}\,+\,{\sum }_{j\in {\{e\}}}{N}_{j}{\mu }_{j})}^{2}$, with carrier densities ${N}_{i,j}$ and mobilities ${\mu }_{i,j}=e{\tau }_{i,j}/{m}_{i,j}$ determined by the carrier relaxation times ${\tau }_{i,j}$ and masses ${m}_{i,j}$ of individual band *i* (of holes) or *j* (of electrons), respectively (Eq. 2.78 of ref. ^23^ with multiple bands). Here, carrier mobilities are expected to have only a weak temperature dependence. On the other hand, when a band moves out from the Fermi level, the relative contributions of charge carriers (electron or hole) dramatically change, signaled by a rapid turn (over a temperature range ~20 K) in the trend followed by *R*_H_(*T*) and similar to other band-gap-opening scenarios exhibited, for example, by charge and spin density waves^24,25^. The first surface moves out from the Fermi level at ~220 K (Fig. 3a, top inset), and with *R*_H_(*T*) turning more electron-like, the departed carriers are of hole type. As thermal excitation at finite temperature still populates the departed bands close to the Fermi level, *R*_H_(*T*) remains a smooth function of *T*. Here we count four characteristic temperatures below *T*_N_—at 220, 165, 80, and 10 K—that reflect four sets of carriers leaving the Fermi level (arrows and *T*_1_−*T*_4_ in Fig. 3a insets). Below *T* ~ 10 K, a sharply divergent *R*_H_ indicates that a full gap emerges in the bulk, and all itinerant carriers are due to thermal excitation, predominately of electron type from the last departed band.

## Discussion

Our results can be compared with existing band structure calculations^26^, adapted here in Fig. 3b, that suggest the Fermi surface is made up of three sets of carriers in the paramagnetic phase of Cd_2_Os_2_O_7_: hole surfaces around the W point, an electron shell at the Γ point, and a family of electron ellipsoids along the Γ−X line, solely of Os 5*d t*_2g_ origin. Our observation reveals a fourth set of hole ellipsoids, potentially located along the Γ−X line^26^ (Fig. 3b), but which have an energy difference too small to be resolved by the band structure calculation given that they drop below the Fermi level at 220 K, only 7 K below *T*_N_. Infrared reflectivity also illuminates the metal-insulator transition in Cd_2_Os_2_O_7_^27,28^. While the direct optical gap has been consistently verified, the interpretation varies from either a spin-density-wave gap opening at *T*_N_^27^ or an indirect gap opening at ~210 K because of a Liftshitz type of mechanism^28^. The latter is partially consistent with our observed first set of carriers departing the Fermi level at 220 K.

Our results demonstrate that the transition at *T*_N_ = 227 K in Cd_2_Os_2_O_7_ should be regarded as only the magnetic-ordering transition, and there is no concurrent metal-insulator transition despite the fact that the resistivity *ρ*(*T*) changes its temperature dependence around *T*_N_ (Fig. 1c inset). Given the well-defined changes observed for *R*_H_(*T*) at *T*_N_ (Fig. 3a), the driving mechanism behind the electronic evolution should be attributed to Os 5*d t*_2g_ band renormalization by the AIAO order^7^, with the effect growing with the increasing strength of the magnetic order parameter, the staggered moment <*m*>, with decreasing *T*. From a direct X-ray magnetic diffraction study^12^, <*m*> continues to grow to the zero-temperature limit without saturation. This spin-dependent shift of the quasiparticles’ self-energy happens within the antiferromagnetic phase and the electronic gap is opened by the developed antiferromagnetic order. It is analogous to a Slater mechanism without Brillouin zone-folding^7^.

The entire density of states within ±1.5 eV of the Fermi level is made of the Os 5*d t*_2g_ manifold of 12 bands in total (two Cd_2_Os_2_O_7_ units in the primitive unit cell, with three *t*_2g_ bands from individual Os ions)^26^. Due to the Os^5+^(5*d*^3^) valence, these bands are filled between the sixth and seventh bands, with dispersion across the Fermi surface to create the electron-type and hole-type carriers. Spin correlation renormalizes the *t*_2g_ manifold to create a true gap at the half level between the sixth and seventh bands.

The Mott−Hubbard picture of the metal-insulator transition splits a single band to open a gap by strong charge correlation energy (*U* ~ 2−6 eV). In Cd_2_Os_2_O_7_, an AIAO antiferromagnet with no structural instability, the transition relies on a small spin-correlation energy (*T*_N_ ~ 20 meV) to essentially create a band insulator. The spin and charge gaps are widely separated in temperature. However, they remain experimentally obscure until the confounding effects of metallic domain wall conduction can be separated from the intrinsic bulk behavior. The methods introduced here clarify the contributions from the bulk and the role of the spin and charge degrees of freedom. They also promise a means to quantify the conductive properties of coercive ferromagnetic domain walls winding their way through an insulating antiferromagnet.

## Methods

### Sample origin and preparation

In this work, we used high-quality Cd_2_Os_2_O_7_ single crystals from ref. ^9^; the vapor transport growth techniques and catalog of physical characteristics are detailed therein. The single crystals used in this work were of octahedral shape and 0.3−0.5 mm in size. Individual crystals were polished to plates of 17−20 μm thickness with a (1, 1, 0) surface normal^14^. Previous synchrotron X-ray magnetic diffraction studies directly verified the quality of these single-crystal plates, demonstrating typical crystal mosaics of 0.01−0.02^o^ FWHM and the existence of AIAO order^14^. Further, the pyrochlore space group *Fd-3m* was verified to the highest sensitivity possible by examining the forbidden orders of (4, 2, 0) and (6, 0, 0) using synchrotron-based single-crystal x-ray diffraction^14^. Samples were selected for these transport measurements from unused plates prepared for the X-ray study, which are of better quality than the sample used in a previous galvanomagnetic study^21^. The sample in ref. ^21^ was cut from a large crystal (3 × 3× 1 mm^3^) with a significantly broader mosaic (supplementary materials of ref. ^21^), and higher density of domain walls, as indicated by a comparison of Fig. 2d in this work to Fig. 3f of ref. ^21^.

### Galvanomagnetic measurements

As the current distribution could potentially change under temperature between the domain walls and bulk, a strict Hall bar-shaped sample geometry is not advantageous, as it could have potentially complex issues in translating from measured resistances to the underlying resistivity. Instead, we employ a van der Pauw (vdP) sample geometry^22^, where the standard conversion depends only on the sample thickness rather than all lateral dimensions. Furthermore, the vdP geometry allows equal access to all combinations of lead configurations in the measurement of both Hall and magnetoresistance (Figs. 1c and 2c insets)^21^. Four 25 μm diameter gold wires were attached to the transport samples of typical 200 μm lateral sizes by conductive silver epoxy.

The transport samples were mounted on the standard sample holder of a horizontal rotator probe in a 14 T Physical Property Measurement System (PPMS, Quantum Design, Inc.). Using the horizontal rotation, we first cool our samples through *T*_N_ with a 2 T magnetic field applied along an in-plane direction *ϕ* (Fig. 2a) then the sample is rotated below *T*_N_ to allow the measurement magnetic field of both the Hall effect and the MR to be applied perpendicular to the sample surface (Fig. 2b). An additional home-built indexing stage on the sample holder provides degree of freedoms to set field-cooling along 24 discrete angular positions of *ϕ* within the sample surface plane^21^; the origin of the *ϕ*-angle has no specific relationship to either the wiring positions of the electrical leads or the crystalline structure. Galvanomagnetic responses of both reciprocal Hall channels and vdP channels were measured at selected temperatures of 195, 30, and 1.8 K for the full *ϕ*-dependence (Fig. 2d−f). The data set at 195K was measured independently from those at the other two temperatures. At 30 and 1.8 K, measurements were repeated at three and eleven *ϕ* positions, respectively, for a check of reproducibility through an additional field-cooling process. For measurements at 30 K, all data except two (one each at *ϕ* = 30^o^ and 195^o^) have a corresponding measurement at 1.8 K during the same field-cooling process in order to compare the *ϕ*-dependence at both temperatures. For the full temperature evolution in Figs. 1, 2g, and 3, both samples were field-cooled along one in-plane *ϕ* position to base temperature; then the galvanomagnetic measurements were performed at each stabilized temperature along the warming trajectory. We observe no degrading or change in our samples after many thermal cycles (>60 in COO-2).

The resistivity was measured using a Lakeshore LS372 AC resistance bridge, working at 9.8 Hz, together with a low-noise 3708 preamp and a home-built vdP switching box based on low-resistance CMOS relay switches. At zero field, the vdP relationship is satisfied to Δ*R*/*R*_max_ = (*R*_Hall1_ + *R*_vdP1_ − *R*_vdP2_)/Max(*R*_vdP1_:*R*_vdP2_) < ±0.05% at 30 K and <±0.25% at 1.8 K. *R*(*H*) curves were measured over a magnetic field loop of ±4 T The coercive field of the ferromagnetic domain walls is known to be >9 T (see, e.g., ref. ^20^), and our measurements over field loops of ±14 T also demonstrate repeatable field dependence. We thus conclude that the domain wall configuration stays constant during each measurement. Since no field hysteresis was observed, *R*(*H*) values are averaged at each field, exemplified by Fig. 2c, and fit to a polynomial form to the second order. The linear slope is taken for plots in Figs. 2 and 3. Our samples demonstrate a very low level of positive parabolic MR ~0.3% over ±14 T, consistent from 1.65 to 80 K, and is thereby unappreciable at low fields. The low level of MR indicates that the cyclotron frequency ${\omega }_{c}$ and carrier relaxation time $\tau$ satisfy ${\omega }_{c}\tau \ll 1$, and the measured Hall coefficient *R*_H_(*T*) is in the low-field limit.

## Data Availability

The data that support the findings of this study are available from the corresponding authors upon reasonable request.
